# Broken tails in Holstein dairy cattle: A cross-sectional study

**DOI:** 10.3168/jdsc.2022-0254

**Published:** 2023-01-02

**Authors:** Hannah E. Olsen, Karly N. Anderson, Katherine C. Creutzinger, Kurt D. Vogel

**Affiliations:** 1 Department of Animal and Food Science, University of Wisconsin–River Falls, River Falls 54022; 2 Department of Animal Science, University of Nebraska–Lincoln, Lincoln 68583

## Abstract

Dairy cows are regularly handled when moved to the milking parlor and during other routine procedures. Low-stress handling methods are important in avoiding negative welfare states for dairy cattle. Tail twisting is used by some handlers to prompt cattle movement. However, when used inappropriately with excessive force, tail twisting can lead to a broken tail. The aim of this cross-sectional study was to determine cow-level factors that may be associated with the prevalence of broken tails in dairy cattle. A subset of 229 Holstein dairy cows (68 primiparous and 161 multiparous) at a single dairy were assessed for broken tails from the larger herd (N = 1,356). Tails were visually assessed for the presence of fractures by a single trained observer. A tail was classified as unfractured if it laid straight when at rest and as fractured if there were deviations in the tail when at rest. Poisson regression models were used to identify associations between cow-level characteristics and broken tails and compute adjusted prevalence ratios (PR). The prevalence of broken tails was 45.8% (105/229) at the time of assessment. Multiparous cows had a greater prevalence of broken tails than primiparous cows [PR = 1.70; 95% confidence interval (CI): 1.11–2.59]. The prevalence of broken tails was also greater for cows treated for mastitis ≥2 times than cows treated once for mastitis (PR = 1.84; 95% CI: 1.08–3.13) and cows never treated for mastitis (PR = 1.36; 95% CI: 1.02–1.82). Results from this study indicated that the longer a cow was present on the farm and the more times she was treated for mastitis, the more likely she was to experience a broken tail. These findings suggest that the relationship between dairy cow handling, health, and welfare is a multifactorial issue.

## Highlights

• A sample of 229 cows from a single herd (N = 1,356) was assessed for broken tails.
• The prevalence of broken tails was 45.8% (105/229) within this herd.
• Multiparous cows had a greater prevalence of broken tails.
• The prevalence of broken tails was greater for cows treated for mastitis ≥2 times.
• Judicious use of tail twisting to prompt movement is important to prevent tail injury.

## Graphical Abstract

**Figure:**
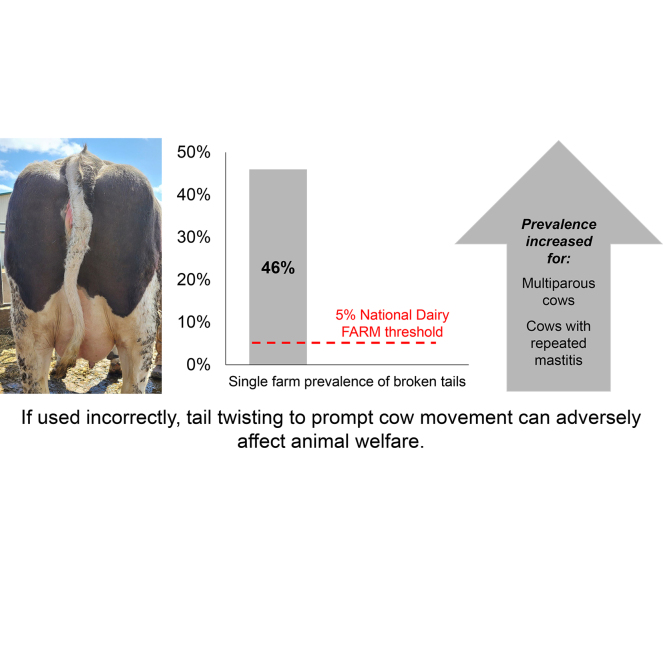
**Summary:** Dairy cattle are handled frequently, and broken tails can result from improper handling. The aim of this crosssectional study was to determine cow-level factors that may be associated with the occurrence of broken tails in dairy cattle. Multiparous cows had a greater prevalence of broken tails than primiparous cows. Cows who were treated for mastitis twice or more had a greater prevalence of broken tails than cows treated once or never. These findings suggest that the longer a cow was present on the farm and the more times a cow was treated for an intramammary infection, the more likely she was to experience a broken tail.

Dairy cows are regularly handled when moved to the milking parlor multiple times per day and during routine management practices. Therefore, low-stress handling methods are important to avoiding negative welfare states for dairy cattle. Tail twisting is used by some handlers to prompt cattle movement. The use of tail twisting as a handling method to prompt forward cattle movement involves releasing the tail after the animal moves forward and never involves continuous twisting of the tail (Validus, 2016). If tail twisting occurs with excessive force, damage to the tail may occur. Damage from excessive force during tail twisting may result in dislocation of the intervertebral joints, tail deviation, and subsequent swelling (Laven, 2020). Broken tails typically do not involve fractures to bones of the tail itself, but rather tears to the caudal fascia and resulting damage to the intervertebral connections (Laven, 2020). Because broken tails do not involve the breaking of the vertebra, a broken tail in this context may be appropriately compared with a dislocated finger (Laven, 2020). Tail injuries can cause substantial pain and distress in dairy cattle (Laven and Jermy, 2020). At this time, limited data have been published regarding the prevalence of broken tails, and even less is known regarding variation in the severity of broken tails in dairy cattle.

Current conjectures regarding the cause of broken tails include facility design (e.g., incorrectly installed automatic manure scrapers) and incorrect handling. It is possible that improper handling, rather than facility design, is the primary cause of broken tails. Laven (2020) anecdotally reported a lack of evidence that facility design contributed to tail breaks on dairies with a high prevalence of broken tails but that inappropriate handling did contribute to broken tails. Tail twisting is a form of operant conditioning that uses negative reinforcement to encourage walking forward but can be an abusive act when not used judiciously. The magnitude of force required to break a tail is unlikely to be accidentally applied by a handler; therefore, the presence of broken tails indicates an issue in animal handling (Laven and Jermy, 2020; National Dairy FARM Program, 2020). Improper or rough handling can have a severely negative impact on dairy cows.

During some animal welfare assessments on dairy farms, tails are assessed as part of the evaluation criteria and are generally considered to be broken or not broken. The National Dairy FARM Program (2020) states that 95% or more of cows should not have a broken tail (i.e., tail swelling or deviation that can be visually observed or the presence of any necrotic tissue). Further, broken tails are described as an easy way to detect injuries inflicted by people (Grandin, 2017). Laven and Jermy (2020) reported that the torque required to break a tail is a least 9.8 N·m (newton-meter) and the maximum torque is 20 N·m; therefore, it is unlikely that sufficient force to break the tail would be accidentally applied when proper handling techniques are used. Although the prevalence of broken tails is not well characterized in the literature, it may indicate the presence or absence of improper handling. There is a lack of published data regarding the causes of broken tails and the welfare implications of a broken tail. The objective of this study was to determine cow-level factors associated with the prevalence of broken tails in dairy cattle.

All procedures involving live animals in this study were approved by the Colorado State University Animal Care and Use Committee. The approved protocol number was 09-175A-01.

A subset of 229 cows from a dairy herd consisting of 1,356 lactating and nonlactating Holstein cows were assessed for locomotion score and fractured tails. The dairy was located in the Western Plains region of the United States. Data for this cross-sectional study were collected during a single visit to the dairy in June 2009. This study was originally designed to assess biochemical markers of energy balance (i.e., nonesterified fatty acid and BHB) and inflammation (i.e., haptoglobin). During blood collection by tail venipuncture, a trained observer noted that many animals had fractured tails. Thus, the prevalence of broken tails was included as an outcome of interest. Cows were included for broken tail analysis if they were scheduled to have blood collected on the final day of data collection (over a 2 d period of data collection).

Cows were housed outdoors in dry lots with gravel and soil substrate without access to shade or stalls. No automatic manure scrapers were present at this facility. A TMR formulated for the appropriate stage of lactation was provided ad libitum by fenceline feed bunks. Ad libitum access to water was also provided within each pen. Animal records were maintained by herd management in DairyComp305 software (Valley Ag software) and transferred to an Excel spreadsheet (Microsoft Corp.).

All dairy cattle were visually assessed for broken tail and locomotion score by a single trained observer. Tails were classified as unfractured if they laid straight when at rest and as fractured if there were any noticeable deviations when at rest. Tails were considered at rest when the entire tail hung directly toward the ground and the switch of the tail was not moving. Our definition of a fractured tail aligned with the definitions of level 2, 3, and 4 tail fractures outlined by Laven (2020). Locomotion scores were assessed using a 5-point scoring system (1 = flat back and all legs bear weight equally; 2 = flat or mildly arched back and slightly asymmetric gait; 3 = arched back and slight limp; 4 = clearly arched back and reluctant to bear weight on at least 1 limb; 5 = extremely arched back and inability to bear weight on 1 or more limbs; Thomsen et al., 2008).

All analyses were performed using SAS software (version 9.4; SAS Institute Inc.). Raw data were visually screened at the individual animal level for data distribution and outliers using the UNIVARIATE procedure in SAS. Statistical significance was declared at *P* < 0.05.

Univariable analysis was conducted between the dependent variable (broken tail) and each explanatory variable using a Poisson regression model (PROC GENMOD). Continuous variables were visually assessed for normality and outliers, and were categorized if they did not meet the assumptions of normality. Parity was categorized into primiparous (parity = 1) and multiparous (parity ≥2). Total lifetime mastitis treatments were categorized into never treated for mastitis, treated for mastitis once, and repeated mastitis treatments (0, 1, and 2, respectively). No outliers were detected in the data set. Variables of interest were tested at the univariable level if they were expected to be biologically relevant to the prevalence of broken tails and included parity, DIM, locomotion score, total lifetime mastitis treatments, and predicted 305-d milk production. Explanatory variables associated with the dependent variable (*P* < 0.20) were included in the multivariable model. Manual backward removal was performed and variables with *P* < 0.05 were retained in the final model. Interactions were tested between biologically relevant variables and removed from the model if *P* > 0.05. Confounding was assessed and a variable was retained in the final model if its removal changed the coefficients by >20%. The prevalence ratio (**PR**) of broken tails (broken vs. unbroken) was analyzed using a Poisson regression model with robust error variances (PROC GENMOD), including a log-link. Standard errors were corrected for overdispersion by adding the “pscale” option to the model statement (Allison, 2001). Cow was included in the repeated statement with an unstructured correlation structure. A Pearson goodness-of-fit test was used to assess the fit of the model with *P* < 0.05.

The study population included 29.7% primiparous and 70.3% multiparous dairy cattle. The population of cows included in the analysis for broken tails was representative of the entire population (Table 1). Multiparous and primiparous cows had 52.8 and 29.4% broken tails, respectively. Prevalence of broken tails was 41.1%, 37.0%, and 68.2% for cows with 0, 1, and 2+ mastitis treatments, respectively. A mastitis treatment was defined as a cow that was treated with antimicrobials for intramammary infections in the herd records.

**Table 1:** Descriptive statistics for the subset of cows selected for broken tail analysis (n = 229) and the entire herd (N = 1,356) of Holstein dairy cows assessed in this cross-sectional study

Attribute	Sample population (n = 229)					Entire population (N = 1,356)			
	n	% of cows in subset	Broken tail, % (no./total)	Average DIM	Predicted 305-d milk yield (kg)	n	% of cows in herd	Average DIM	Predicted 305-d milk yield (kg)
Parity									
Primiparous	68	29.7	29.4 (20/68)	165.9	18,128.8	532	39.2	223.7	17,798.6
Multiparous	161	70.3	52.8 (85/161)	183.7	22,078.2	824	60.8	214.6	22,047.2
Mastitis treatments per cow									
0	158	69.0	41.1 (65/158)	178.5	20,214.0	997	73.5	214.9	19,754.6
1	27	11.8	37.0 (10/27)	186.6	23,069.3	133	9.8	218.2	22,507.8
2+	44	19.2	68.2 (30/44)	173.3	22,109.5	226	16.7	220.4	22,068.2

The objective of this study was to evaluate cow-level factors associated with broken tails in dairy cattle. In this study, we found that multiparous cows had a greater PR of broken tails than primiparous cows. Multiparous cows are at an increased risk for culling for a variety of reasons, including infertility, mastitis, skeletal injuries, and lameness (Brickell and Wathes, 2011). Older animals have a greater accumulation of life experiences, which may reveal concerns at the farm level that would not be observed in younger animals.

Cows with repeated mastitis treatments also had a greater PR of broken tails than cows treated once or never treated for mastitis (Table 2). Of the primiparous cows included in the subset, none had been treated for mastitis at the time of data collection. For this reason, we were unable to evaluate the interaction between mastitis treatments and parity. Cows treated for mastitis ≥2 times had a greater PR of broken tails than cows with a single treatment or cows that had never been treated for mastitis. We speculate that this finding may be due to a cow's reluctance to enter the milking parlor during or after an intramammary infection. Cows with intramammary infections experience udder pain, which can be increased during udder manipulation (Banting et al., 2008; Peters et al., 2015). For example, Banting et al. (2008) found that cows had an increase in pain sensitivity at udder palpation following experimental induction of mastitis. Thus, it is likely that cows with mastitis experience pain during milking. Medrano-Galarza et al. (2012) found that cows had a higher frequency of kicking, lifting, and stepping during milking during the first 3 d after an intramammary infection was detected. The authors concluded that the changes in behavior during milking were pain behaviors due to the presence of mastitis. Cows avoid areas that they perceive negatively (Grandin et al., 1994; Pajor et al., 2000). The pain-based memories for cows who have previously experienced mastitis associated with the milking parlor may increase their reluctance to enter. Thus, the force used by farm workers to move these cows into the parlor may increase. This explanation, however, is speculative, and further research is required to understand cows' reluctance to enter the parlor in relationship with mastitis treatment history or handling practices.

**Table 2:** Explanatory variables identified as predictors of broken tails in Holstein dairy cows (n = 229)^1^

Dependent variable	Prevalence ratio	95% CI		*P*-value
		Lower	Upper	
Parity				
Multiparous vs. primiparous	1.70	1.11	2.59	0.014
Mastitis treatments per cow				
2+ vs. 1	1.84	1.08	3.13	0.025
2+ vs. 0	1.36	1.02	1.82	0.035
1 vs. 0	0.74	0.43	1.26	0.270

1 Cows were categorized as having a broken (n = 105) or unbroken tail (n = 124) in a cross-sectional study.

Broken tails are included as an animal-based measure of dairy welfare assessments (Validus, 2016; National Dairy FARM Program, 2020). At the dairy in the current study, 46% of cows assessed for broken tails had broken tails, which is greater than the 5% of broken tails permitted under the National Dairy FARM Program. This evaluation benchmark is part of the National Dairy FARM Program's animal-based measures, and failure to meet the benchmark results in the farm being issued a Continuous Improvement Plan, with improvements expected within the next 3 yr. The large percentage of cows with broken tails in our study indicates that the problem of broken tails was under-addressed, and further investigations are needed to determine the causes behind broken tails as well as management techniques to help prevent tails from being broken. Animal handling techniques were not investigated in this study; therefore, we cannot determine whether the broken tails were due to poor or abusive handling.

A primary limitation of our study is that a cross-sectional sample of a single dairy was observed, and only a subset of the animals in this herd were assessed for broken tails; the result of this was a relatively small sample size. Only cow-level factors could be evaluated; as such, facility and management factors could not be assessed. Because animal handling was not evaluated, the frequency of tail twisting is unknown. Additionally, the facility design of this dairy may not be generalizable to dairies outside of the Western Plains region of the United States, where this study took place. Since the time of data collection, guidance has indicated that palpation of the tail should occur during assessments of tail damage (DairyNZ, 2022). It is also possible the prevalence of broken tails was underestimated in this study because tail assessments were visual and did not include palpation of the tail.

This study aimed to identify cow-level factors associated with the occurrence of broken tails on dairy farms. Multiparous cows had a greater prevalence of broken tails than primiparous cows. Cows with repeated mastitis treatments were also more likely to have a broken tail than cows treated once or never treated for mastitis. There are still gaps in our knowledge considering the prevalence and implications of broken tails in dairy cattle. Further research is needed to deepen our understanding of how and why tails are broken, the welfare implications of broken tails, and how broken tails can be prevented on dairies. Future studies could include quantifying the occurrence of tail twisting to prompt forward movement on dairies or the occurrence of forceful tail twisting on dairies and how often this technique results in a broken tail.
